# Highly Selective Tau-SPECT Imaging Probes for Detection of Neurofibrillary Tangles in Alzheimer’s Disease

**DOI:** 10.1038/srep34197

**Published:** 2016-09-30

**Authors:** Masahiro Ono, Hiroyuki Watanabe, Ayane Kitada, Kenji Matsumura, Masafumi Ihara, Hideo Saji

**Affiliations:** 1 Department of Patho-Functional Bioanalysis, Graduate School of Pharmaceutical Sciences, Kyoto University, 46-29 Yoshida Shimoadachi-cho, Sakyo-ku, Kyoto 606-8501, Japan; 2 Department of Stroke and Cerebrovascular Diseases, National Cerebral and Cardiovascular Center, 5-7-1 Fujishiro-dai, Suita, Osaka 565-8565, Japan

## Abstract

Neurofibrillary tangles composed of aggregates of hyperphosphorylated tau proteins are one of the neuropathological hallmarks of Alzheimer’s disease (AD) in addition to the deposition of β-amyloid plaques. Since the deposition of tau aggregates is closely associated with the severity of AD, the *in vivo* detection of tau aggregates may be useful as a biomarker for the diagnosis and progression of AD. In this study, we designed and synthesized a new series of radioiodinated benzoimidazopyridine (BIP) derivatives, and evaluated their utility as single photon emission computed tomography (SPECT) imaging agents targeting tau aggregates in AD brains. Five radioiodinated BIP derivatives were successfully prepared in high radiochemical yields and purities. In *in vitro* autoradiographic studies using postmortem AD brains, all BIP derivatives displayed high accumulation of radioactivity in the brain sections with abundant neurofibrillary tangles, while no marked radioactivity accumulation was observed in the brain sections with only β-amyloid aggregates, indicating that the BIP derivatives exhibited selective binding to tau aggregates. Biodistribution studies in normal mice showed high brain uptake at 2 min postinjection (3.5–4.7% ID/g) and rapid clearance at 60 min postinjection (0.04–0.23% ID/g), which is highly desirable for tau imaging agents. The results of the present study suggest that [^123^I]BIP derivatives may be useful SPECT agents for the *in vivo* imaging of tau aggregates in AD.

Alzheimer’s disease (AD), characterized by declines in memory and recognition, constitutes a representative neurodegenerative disorder. As the population ages throughout the world, a rapid increase in the number of patients with AD is a concern. However, the early and definitive diagnosis of AD is difficult solely based on an interview or a conventional diagnostic imaging technique, including measurements of a glucose metabolism or cerebral blood flow with nuclear medical imaging techniques, such as positron emission tomography (PET) or single photon emission computed tomography (SPECT)^1^. Therefore, the establishment of a diagnostic imaging technique that can diagnose AD precisely is strongly desired. The pathological changes in the AD brain include senile plaques and neurofibrillary tangles, which are formed by aggregates of β-amyloid (Aβ) peptides and hyperphosphorylated tau proteins, respectively^2^. Therefore, these pathological changes in the brains represent important targets for the diagnosis and therapy of AD^3^,^4^. It is well known that the nuclear medical imaging methods including PET and SPECT with imaging probes labeled with radionuclides can noninvasively detect *in vivo* biomarkers from the outside of the body by utilizing the high permeability of radioactive rays emitted from imaging probes. It is therefore expected that PET and SPECT will become applicable for the *in vivo* imaging of Aβ plaques and neurofibrillary tangles. Accordingly, a number of PET and SPECT imaging probes have been actively developed. On the basis of the amyloid cascade hypothesis that the deposition of Aβ plaques is the causative agent of AD^5^, the development of Aβ imaging probes that can detect Aβ plaques *in vivo* has been extensively carried out^6^,^7^,^8^. However, many clinical studies with these Aβ imaging probes revealed that there were a number of false-positive subjects who did not develop AD even though Aβ imaging displayed the existence of numerous Aβ plaques in their brains^9^,^10^,^11^. Therefore, it has been recognized that the highly precise diagnosis of AD only by Aβ imaging may be difficult.

In contrast, it has been reported that tau aggregates develop similarly to Aβ before the development of AD, and there is a close correlation between tau accumulation and clinical manifestations^12^,^13^. Therefore, it has been generally accepted that tau imaging may be effective for not only the early detection of AD but also the judgment of its progression^13^,^14^,^15^. Currently, the development of tau imaging probes for PET is actively progressing. Among them, [^18^F]THK-5117^16^,^17^, [^18^F]THK-5351^18^, [^18^F]T807^19^,^20^, and [^11^C]PBB3^21^,^22^ are the tau-PET imaging probes showing the most promise in clinical studies. Although the number of PET facilities is limited in the world, SPECT is more convenient than PET with regards to the number of facilities and its use of radionuclides with a longer half-life than PET. In order to diagnose many more AD patients including preclinical AD patients, the development of a tau-SPECT imaging method is important in addition to the tau-PET imaging method. However, there has been no report on a useful tau imaging probe with radionuclides for SPECT. We have developed tau imaging probes for SPECT, and have reported such probes in our recent papers^23^,^24^,^25^,^26^.

In order to image tau aggregates *in vivo*, requirements for tau imaging probes primarily include selective binding affinity for tau aggregates and high uptake into and rapid clearance from the brain at an early time postinjection^13^,^15^. Through our recent structure-activity relationship studies of heterocyclic phenylethenyl and pyridinylethenyl derivatives for the development of tau imaging probes for SPECT^26^, we found that one of the phenylethenyl benzimidazole derivatives (SBI-2, Fig. 1A) may be a promising tau imaging probe. Although this compound served as a tau imaging probe with regard to relatively selective binding to tau aggregates against Aβ aggregates and favorable pharmacokinetics in the brain, it did not show sufficient binding affinity to tau aggregates to obtain clear images^26^. In addition, we observed photoisomerization of the ethenyl group in the molecule of SBI-2. To prevent the photoisomerization, in this study, we newly designed a phenylbenzimidazopyridine (BIP-Ph), which is formed by cyclization of SBI-2 (Fig. 1A). Moreover, we designed BIP-H which is produced by removing the phenyl group of BIP-Ph, and BIP-Me, BIP-OMe, and BIP-NMe_2_, which are produced by the introduction of a methyl group, methoxy group, and dimethyamino group into BIP-H, respectively (Fig. 1A). After the synthesis of five novel BIP derivatives, we evaluated their utility as tau imaging probes for SPECT from the viewpoint of the selective binding affinity and uptake into and clearance from the brain. This is the first report on the development of tau-SPECT imaging probes based on the radioiodinated BIP scaffold.

## Results and Discussion

### Chemistry

**Figure 1:**
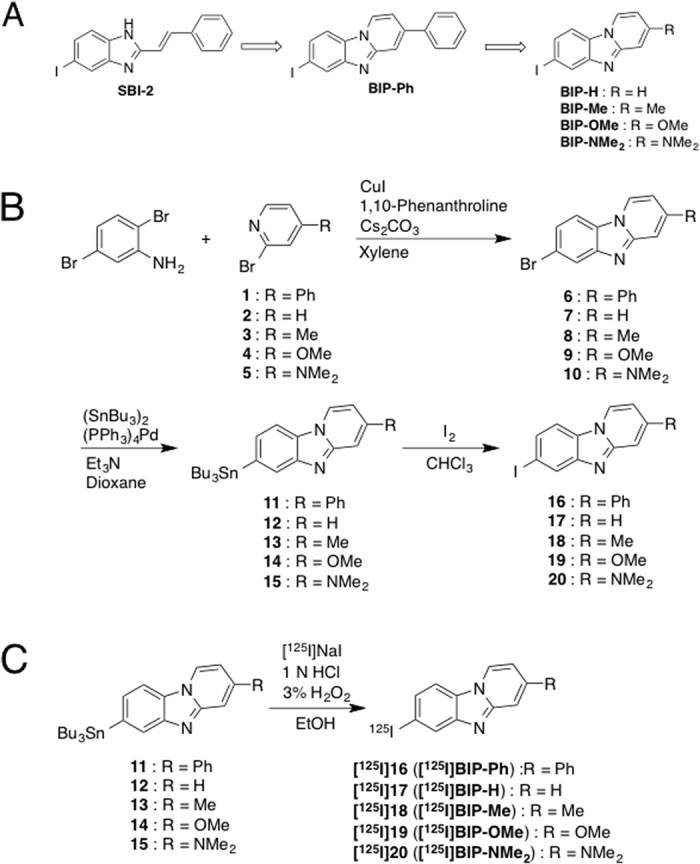
(**A**) Flow of the development of tau-SPECT imaging probes based on the BIP scaffold. (**B**) Synthetic route of BIP derivatives. (**C**) Radioiodination reaction of BIP derivatives.

We carried out the synthesis of BIP derivatives according to Fig. 1B. The starting materials (**1** and **5**) for BIP-Ph^27^ and BIP-NMe_2_^28^ were synthesized according to the method reported previously. The other starting 2-bromopyridine derivatives used were commercially available products. The BIP scaffold was prepared by the reaction of various 2-bromopyridine derivatives with 2,5-dibromoaniline in the presence of 1,10-phenanthroline and cesium carbonate according to a method reported previously^29^ to yield target bromo compounds (**6**, **7**, **8**, **9**, and **10**). After the reaction for the formation of the BIP scaffold, the tributyltin derivatives (**11**, **12**, **13**, **14**, and **15**) were prepared from the corresponding bromo compounds using a conventional bromo to tributyltin exchange reaction catalyzed by Pd(0). The tributyltin derivatives were reacted with I_2_ in chloroform at room temperature to yield the iodo compounds (**16**, **17**, **18**, **19**, and **20**). The tributyltin derivatives were also used as a precursor for labeling with ^125^I. In the present study, we used ^125^I as radioiodine instead of ^123^I because of its availability and longer half-life. Radioiodination of BIP derivatives was performed by the iododestannyaltion reaction from the corresponding tributyltin derivatives using hydrogen peroxide as the oxidant (Fig. 1C). Then, we obtained radioiodinated BIP derivatives in 30–65% radiochemical yields and over 99% radiochemical purities after HPLC purification.

### *In vitro* autoradiography using AD brain sections

In recent papers regarding the development of tau imaging probes, several methods to evaluate the selective binding affinity for tau aggregates have been reported^17^,^19^,^21^. Among them, we selected an *in vitro* autoradiographic (ARG) study using brain sections from AD patients, because an *in vitro* ARG study enables more direct evaluation than an *in vitro* binding assay using aggregates of recombinant tau proteins. When we performed immunohistochemical staining of two kinds of AD brain sections (frontal and temporal lobes) with anti-Aβ antibody and anti-phosphorylated tau antibody, we could observe the extensive accumulation of Aβ plaques in the gray matter of the frontal lobe, but not tau aggregates (Figure S1A and B). In contrast, the gray matter of the temporal lobe showed marked accumulation of both Aβ plaques and tau aggregates in a different pattern of immunohistochemical staining (Figure S1C and D). Therefore, in the case that we use these two kinds of AD brain sections, ideal tau imaging probes should specifically accumulate only in the temporal lobe positive for both tau and Aβ and not accumulate in the frontal lobe negative for tau. Also, *in vitro* autoradiography of each probe should correspond to the immunohistochemical staining of tau. On the basis of these criteria, we carried out *in vitro* autoradiography from the perspective of these evaluations.

**Figure 2:**
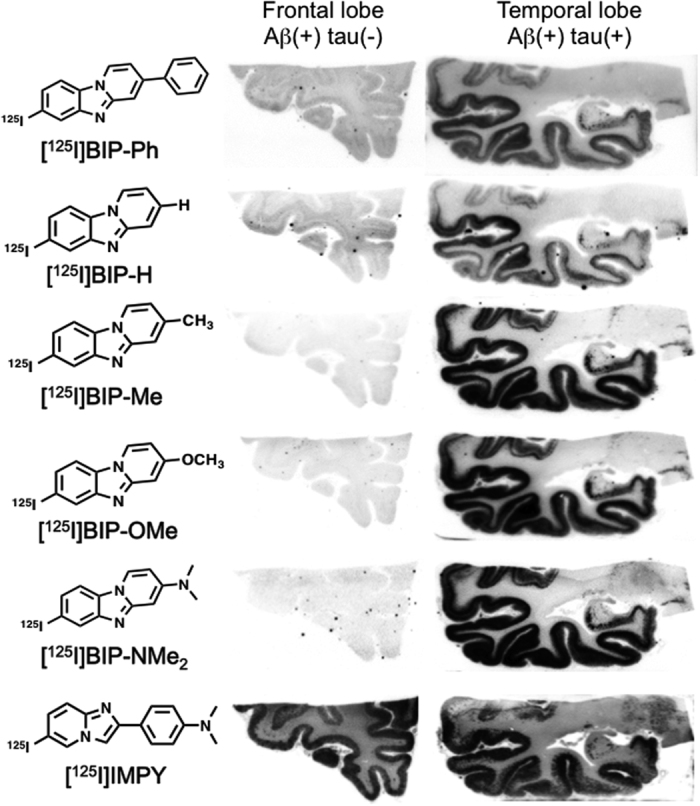
Comparison of *in vitro* autoradiograms with ^125^I-labeled BIP derivatives in AD brain sections from the frontal and temporal lobes. *In vitro* autoradiography with [^125^I]IMPY was also performed.

**Figure 3:**
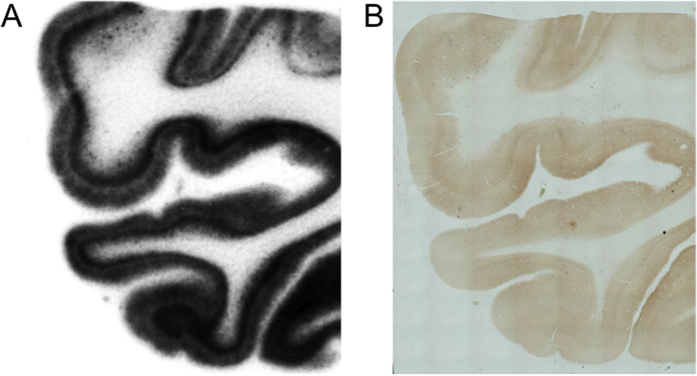
Magnified image of an *in vitro* autoradiogram with [^125^I]BIP-NMe_2_ (**A**) and immunohistochemical staining of the adjacent brain section with anti-phosphorylated tau antibody (**B**).

**Figure 4:**
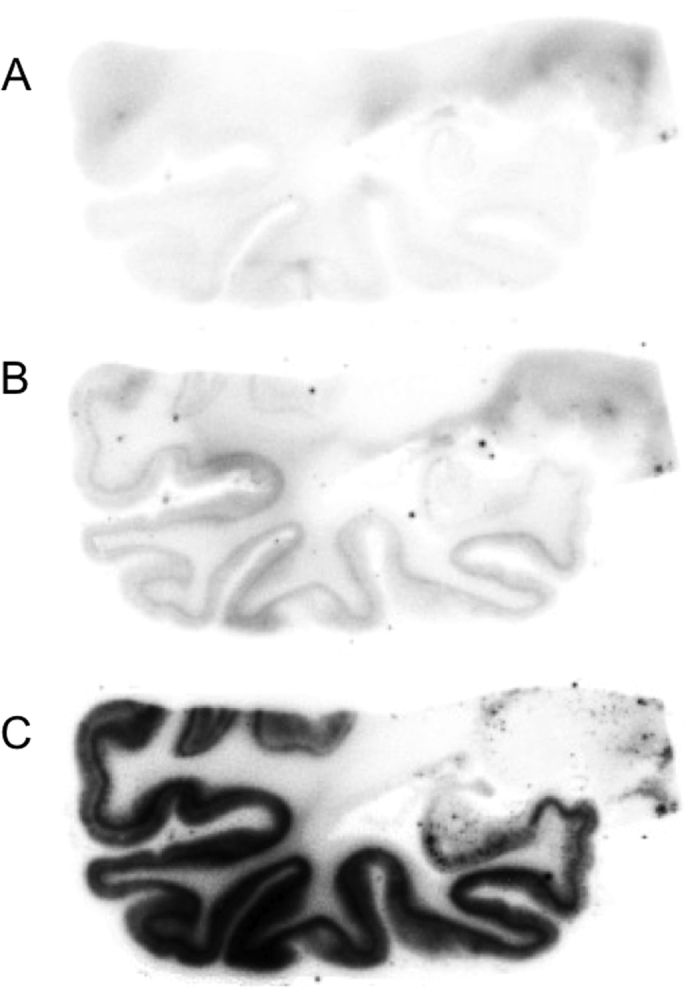
*In vitro* autoradiograms of [^125^I]BIP-NMe_2_ on AD brain sections with excess BIP-NMe_2_ of 10 μM (**A**) and 1 μM (**B**) or without BIP-NMe_2_ (**C**).

Figure 2 shows the results of the *in vitro* autoradiography of [^125^I]BIP derivatives and [^125^I]IMPY. When we performed optimal adjustment of the autoradiograms for each probe, all BIP derivatives showed a similar pattern on *in vitro* autoradiography, and the marked radioactivity accumulation was specifically observed in the gray matter of the temporal lobe. In contrast, no marked radioactivity accumulation was found in the gray matter of the frontal lobe, which does not contain a tau pathology. As we mentioned above, the results of immunohistochemical staining suggested that there are both Aβ plaques and neurofibrillary tangles in the gray matter of the temporal lobe, while only Aβ plaques mainly deposit in the gray matter of the frontal lobe. This pattern was completely different from that of [^125^I]IMPY, which is well known as a SPECT imaging probe for Aβ plaques^30^,^31^. [^125^I]IMPY showed marked binding to Aβ plaques in the gray matter of both the frontal and temporal lobes. Figure S2 shows comparative enlarged autoradiograms of [^125^I]IMPY and [^125^I]BIP-NMe_2_. The radioactivity of the autoradiogram of [^125^I]IMPY was seen as numerous spots reflecting the binding to Aβ plaques, while that of [^125^I]BIP-NMe_2_ was observed as the laminar accumulation characteristic of neurofibrillary tangles. These results strongly suggest that all BIP derivatives may bind to tau aggregates selectively. Figure 3 shows a representative enlarged image of an *in vitro* ARG study using BIP-NMe_2_ together with that of immunohistochemical staining with anti-phosphorylated tau antibody. BIP-NMe_2_ displayed laminar radioactivity accumulation along the gray matter characteristic of tau accumulation, suggesting that BIP derivatives could clearly detect the tau pathology. Furthermore, to determine whether the radioactivity accumulation of [^125^I]BIP-NMe_2_ in the gray matter of the temporal lobe is specific to tau aggregates, we carried out *in vitro* autoradiographic studies in the presence of excess nonradioactive BIP-NMe_2_ at 1 and 10 μM (Fig. 4). As a result, the high radioactivity accumulation of [^125^I]BIP-NMe_2_ in the brain section was markedly reduced by adding excess nonradioactive BIP-NMe_2_, indicating that [^125^I]BIP-NMe_2_ can specifically bind to tau aggregates in the brain (Fig. 4).

### Quantitative analysis of autoradiography (ARG)

**Figure 5:**
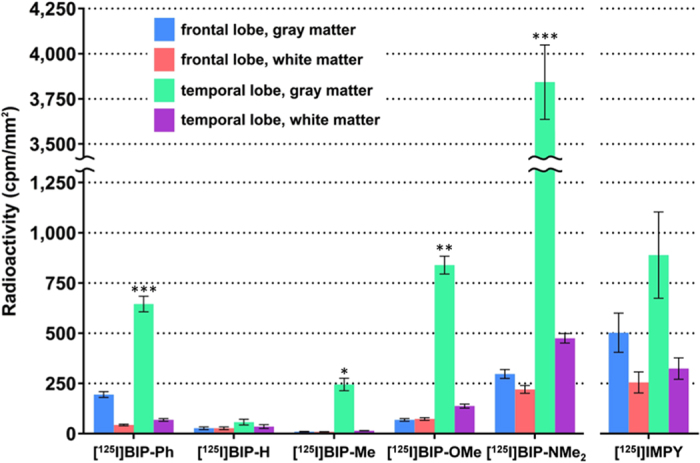
Quantitative analysis of *in vitro* autoradiography with AD brain sections. Data are presented as mean ± SEM (n = 3–7). Statistical significance was analyzed between temporal lobe and frontal groups in gray matter using unpaired t-test, with Welch’s correction as necessary (*p < 0.05, **p < 0.01, ***p < 0.001).

Next, we conducted quantitative analyses of *in vitro* autoradiographic studies as described above. The regions of interest (ROIs) were set in the gray matter of the frontal lobe, white matter of the frontal lobe, gray matter of the temporal lobe, and white matter of the temporal lobe, respectively, in the ARG images (n = 3–7), and the radioactivity accumulation (cpm/mm^2^) in each region was determined for each probe (Fig. 5). As a result, all BIP derivatives showed higher radioactivity accumulation in the gray matter of the temporal lobe, in which abundant tau aggregates exist, than any other regions. This tendency was marked for BIP-Ph, BIP-Me, BIP-OMe, and BIP-NMe_2_ other than BIP-H. When we performed statistical analyses to compare the radioactivity accumulation in the gray matter between the frontal and temporal lobes, BIP-Ph, BIP-Me, BIP-OMe, and BIP-NMe_2_ showed significantly higher radioactivity levels than BIP-H. This also suggests that the substituted group in the BIP scaffold may play an important role in the selective binding affinity to tau aggregates. To further evaluate the selective binding between tau and Aβ, we compared the radioactivity accumulation observed in the gray matter of the frontal lobe with only Aβ aggregates with that observed in the gray matter of the temporal lobe with both Aβ and tau aggregates. When we calculated the ratio of the radioactivity accumulation in the gray matter of the temporal lobe against the gray matter of the frontal lobe, the values varied with the kind of the substituted group introduced into the BIP scaffold (Table 1). All BIP derivatives showed higher radioactivity accumulation in the gray matter of the temporal lobe than that in the gray matter of the frontal lobe. In particular, the BIP derivatives (BIP-Me, BIP-OMe, and BIP-NMe_2_) with a compact substituted group showed a higher ratio than BIP-H which has no substituted group. Although BIP-Ph showed marked accumulation in the tau pathology, the selective binding of BIP-Ph in the τ pathology (3.3) was lower than that of BIP-Me (23.9), BIP-OMe (12.2), and BIP-NMe_2_ (12.9), because BIP-Ph also showed relatively high-level binding to Aβ plaques. Although IMPY also showed a ratio of 1.8, this value may reflect the ratio of Aβ aggregates deposited in the frontal and temporal lobes, and not tau aggregates. These findings in the *in vitro* autoradiographic studies revealed that the selective binding of BIP derivatives to tau aggregates can be markedly increased by introducing the compact substituted group into the BIP scaffold.

### Brain uptake and clearance

**Figure 6:**
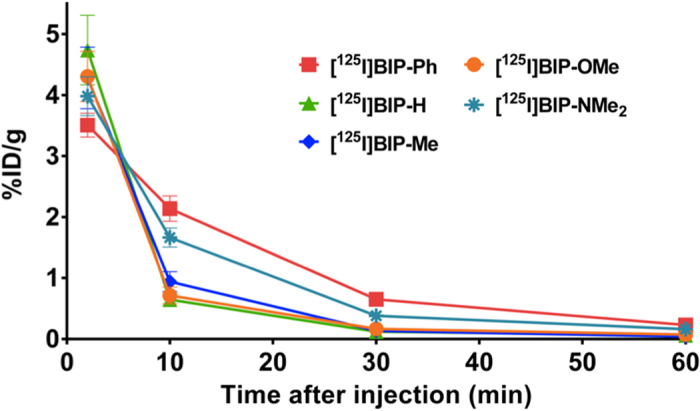
Comparison of uptake into and clearance from the brain after intravenous injection of ^125^I-labeled BIP derivatives into normal mice (n = 5).

Next, we evaluated uptake into and clearance from the brain after the injection of BIP derivatives into normal mice (Fig. 6 and Table 2). In order to obtain highly specific signals of tau aggregates, favorable tau imaging probes need to show not only high brain uptake early after administration but also rapid clearance from the brain because no tau aggregates exist in the brains of normal mice. We determined the log P-values of the ^125^I-labeled BIP derivatives, and the values ranged from 2.64 to 3.37. A previous report suggested that the low-molecular-weight compounds, which have moderate log P-values ranging from 1 to 3, sufficiently penetrate the blood-brain barrier^32^. Since the BIP derivatives showed moderate lipophilicity close to the appropriate log P-values reported in the previous paper, they were expected to penetrate the blood-brain barrier. All BIP derivatives displayed high brain uptake, ranging from 3.5 to 4.7% ID/g, at 2 min postinjection. Thereafter, the radioactivity in the brain cleared with time, and it decreased to 0.12–0.65% ID/g at 30 min postinjection and 0.04–0.23% ID/g at 60 min postinjection. When we calculated the ratio of the radioactivity accumulation of the BIP derivatives, the values ranged from 5 to 40 for 2 min/30 min and from 15 to 104 for 2 min/60 min, indicating that the BIP derivatives showed rapid clearance from the brain. Initial brain uptake of the BIP derivatives at 2 min postinjection were well correlated with their molecular weight, and not their log P-values. In other words, as the molecular weight of the BIP derivatives decreased, they showed higher brain uptakes. In particular, BIP-H and BIP-Me with log P-values with over 3 showed faster clearance from the brain than other BIP derivatives with log P-values less than 3. In addition, the BIP derivatives with a higher molecular weight tended to be cleared from the brain faster in comparison with the BIP derivatives with a lower molecular weight. Previous studies reported that the maximum brain uptake of [^11^C]PBB3^22^, [^18^F]THK-5351^18^, and [^18^F]T807^19^, which are tau-PET imaging probes used in clinical research, showed 1.92, 4.35, and 4.43% ID/g at 1 or 2 min postinjection, respectively. Thereafter, the radioactivity accumulated in the brain cleared with time, and it was reduced to 0.11, 0.21, and 0.62% ID/g at 30 min postinjection for [^11^C]PBB3, [^18^F]THK-5351, and [^18^F]T807, respectively. The ratio of the radioactivity accumulation at 1 or 2 min, and 30 min postinjection was 17.5, 20.7, and 7.15 for [^11^C]PBB3, [^18^F]THK-5351, and [^18^F]T807, respectively. A comparison of these values revealed that some of the radioiodinated BIP derivatives have superior radioactivity pharmacokinetics in the brain. The findings suggest that the BIP derivatives may be applicable to clinical use from the viewpoint of radioactivity pharmacokinetics in the brain, in addition to highly selective binding to tau aggregates. The biodistribution of BIP derivatives in the whole mouse body displayed typical pharmacokinetics of lipophilic compounds, which means high uptake into the liver and the subsequent excretion into the intestine (Table S1). In addition, the low accumulation of BIP derivatives in the thyroid also demonstrated high stability against deiodination *in vivo*.

**Figure 7:**
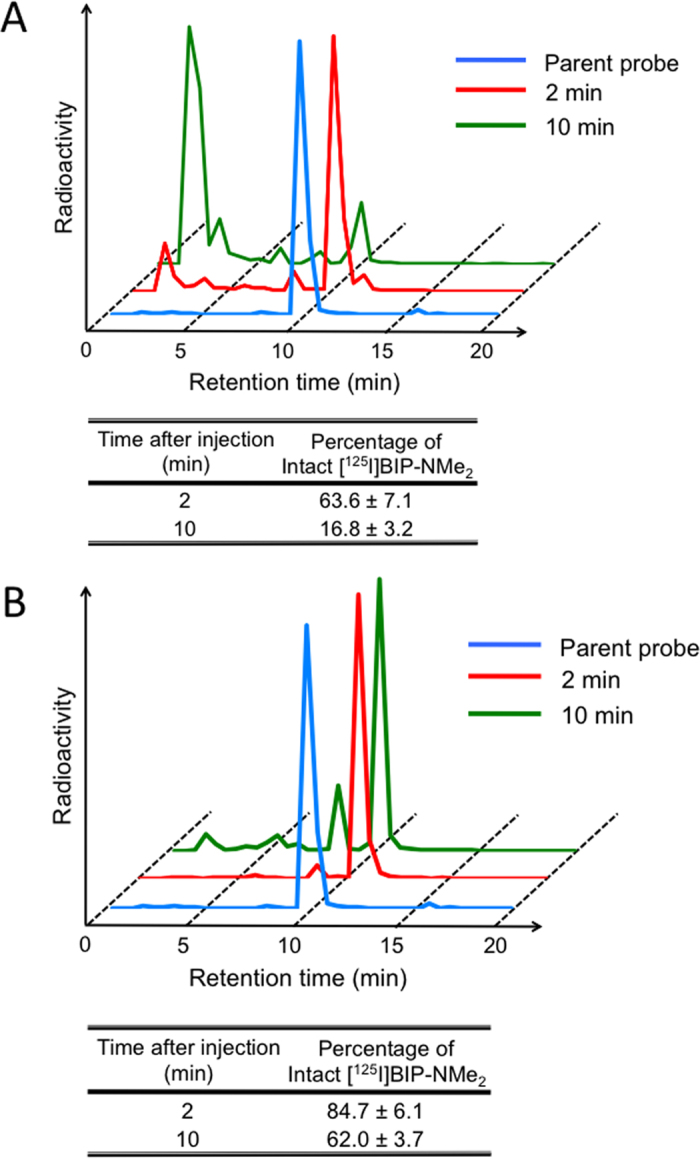
HPLC analysis of radioactivity in the blood (**A**) and brain (**B**) after injection of [^125^I]BIP-NMe_2_ into mice (n = 4).

When we determined the stability of [^125^I]BIP-NMe_2_ in the blood by HPLC analyses, the HPLC profile after incubation for 60 min was similar to that of the intact form, suggesting that they showed high stability in the blood (Figure S3). Furthermore, we analyzed the radiometabolites in the blood and brain after the injection of [^125^I]BIP-NMe_2_ into normal mice. When the radioactivity level was analyzed in plasma by HPLC after the injection of [^125^I]BIP-NMe_2_ into normal mice, we found that [^125^I]BIP-NMe_2_ converted to some radiometabolites with different chemical forms (Fig. 7A). The percent of the intact form decreased with time and reached 63.6 and 16.8% at 2 and 10 min after the injection, respectively. This result regarding *in vivo* metabolism in the blood was comparable to that of [^18^F]THK-5351 reported in a previous paper^33^, suggesting that [^125^I]BIP-NMe_2_ has *in vivo* stability sufficient for clinical trials. The difference in the stability of [^125^I]BIP-NMe_2_ between *in vitro* and *in vivo* may be involved in the different characteristics of radiometabolites formed by the *in vivo* metabolism of [^125^I]BIP-NMe_2_ in organs including the liver and kidney. When we analyzed the radioactivity in the brain by HPLC at 2 and 10 min postinjection of [^125^I]BIP-NMe_2_, 84.7 and 62.0% of [^125^I]BIP-NMe_2_ at 2 and 10 min postinjection, respectively, existed in the brain as an intact form (Fig. 7B), indicating that [^125^I]BIP-NMe_2_ may be sufficiently applicable for the in vivo imaging of tau aggregates. It is generally accepted that the *in vivo* metabolism of radiotracers is different between mice and human subjects. Therefore, further investigation of the characteristics of radiometabolies derived from BIP derivatives in human subjects is essential for clinical application.

## Conclusions

In the present study, we newly designed and synthesized radioiodinated BIP derivatives with various substituted groups, and evaluated their utility as tau-SPECT imaging probes. In *in vitro* autoradiographic studies using AD brain sections, the BIP derivatives depicted tau clearly. In addition, the *in vitro* autoradiographic studies also revealed highly selective binding of the BIP derivatives to the tau pathology compared with Aβ pathology. In the biodistribution study in normal mice, all BIP derivatives showed high uptake into and fast clearance from the brain comparable to the tau-PET imaging probes in clinical use. The results of the present study suggest that ^123^I-labeled BIP derivatives with highly selective affinity for tau aggregates may be new candidates for *in vivo* detection of the tau pathology.

## Materials and Methods

### General remarks

All reagents were commercial products and used without further purification unless indicated otherwise. All compounds were purified by Smart Flash EPCLC W-Prep 2XY (Yamazen corporation) unless indicated otherwise. ^1^H NMR spectra were recorded on a JNM-ECS400 (JEOL) with tetramethyl silane (TMS) as an internal standard. Coupling constants are reported in Hertz. Multiplicity was defined as singlet (s), doublet (d), or multiplet (m). ESI mass spectrometry was conducted with a SHIMADZU LCMS-2020. High-resolution mass spectrometry (HRMS) was carried out with a GCMate II (JEOL). HPLC was performed with a Shimadzu system (an LC-20AT pump with an SPD-20A UV detector, λ = 254 nm) using a Cosmosil C_18_ column (Nacalai Tesque, COSMOSIL 5C_18_-AR-II 4.6 mm I.D. × 150 mm) and acetonitrile/H_2_O (with or without TFA) as the mobile phase at a flow rate of 1.0 mL/min. All key compounds were proven by this method to show >95% purity.

### Animals

Animal experiments were conducted in accordance with our institutional guidelines and were approved by the Kyoto University Animal Care Committee. Male ddY mice were purchased from Japan SLC, Inc. (Shizuoka, Japan), and were fed standard chow and had free access to water. We made all efforts to minimize suffering.

### Human brain tissues

Experiments involving human subjects were performed in accordance with relevant guidelines and regulations and were approved by the ethics committee of Kyoto University. Informed consent was secured from all subjects in this study. Postmortem brain tissues from an autopsy-confirmed case of AD (male, 76 years old) were obtained from the Graduate School of Medicine, Kyoto University.

### Chemistry

Compounds **1** and **5** were synthesized according to a method reported previously^27^,^28^. We used compounds **2**, **3**, and **4** of commercially available products.

### 7-Bromo-3-phenylpyrido[1,2-*a*]benzimidazole (6)

A mixture of **1**^27^ (645 mg, 2.75 mmol), 2,4-dibromopyridine (690 mg, 2.75 mmol) and CuI (105 mg, 0.550 mmol), Cs_2_CO_3_ (2.67 g, 8.26 mmol), and 1,10-phenanthroline (198 mg, 1.10 mmol) in xylene (30.0 mL) was stirred under reflux for 24 h. The mixture was extracted with ethyl acetate (100 mL), and the organic phase was separated and dried over MgSO_4_. The solvent was removed and the residue was purified by silica gel chromatography (ethyl acetate/hexane = 1/2) to give 78.4 mg of **6** (8.80%). ^1^H NMR (400 MHz, CDCl_3_) δ 8.47 (d, *J* = 7.2 Hz, 1H), 8.08 (s, 1H), 7.88 (s, 1H), 7.77 (d, *J* = 8.7 Hz, 1H), 7.72 (d, *J* = 7.2 Hz, 2H), 7.45–7.55 (m, 4H), 7.19 (d, *J* = 7.0 Hz, 1H). ^13^C NMR (100 MHz, CDCl_3_) δ 146.4, 142.9, 141.4, 138.1, 129.2, 129.1, 127.0, 125.0, 124.1, 122.5, 119.0, 114.4, 111.5, 111.01, 110.97. MS (ESI) *m/z* 323.1 [MH^+^].

### 7-Bromopyrido[1,2-*a*]benzimidazole (7)

The same reaction described above to prepare **6** was used, and 834 mg of **7** was obtained in a yield of 67.8%. ^1^H NMR (400 MHz, DMSO-*d*_6_) δ 9.12 (d, *J* = 6.7 Hz, 1H), 8.31 (d, *J* = 8.4 Hz, 1H), 8.01 (d, *J* = 1.5 Hz, 1H), 7.69 (d, *J* = 9.3 Hz, 1H), 7.60–7.64 (m, 1H), 7.52 (dd, *J* = 8.7, 1.7 Hz, 1H), 7.06 (dd, *J* = 6.7, 6.7 Hz, 1H). ^13^C NMR (100 MHz, CDCl_3_) δ 149.1, 145.6, 129.9, 127.5, 125.1, 124.0, 122.5, 118.9, 118.0, 111.5, 110.8. MS (ESI) *m/z* 247.1 [MH^+^].

### 7-Bromo-3-methylpyrido[1,2-*a*]benzimidazole (8)

The same reaction described above to prepare **6** was used, and 62.4 mg of **8** was obtained in a yield of 23.9%. ^1^H NMR (400 MHz, CDCl_3_) δ 8.28 (d, *J* = 7.0 Hz, 1H), 8.02 (s, 1H), 7.70 (d, *J* = 8.7 Hz, 1H), 7.40–7.43 (m, 2H), 6.72 (d, *J* = 7.0 Hz, 1H), 2.48 (s, 3H). ^13^C NMR (100 MHz, CDCl_3_) δ 149.6, 145.8, 141.5, 127.5, 124.1, 123.3, 122.1, 118.5, 115.9, 113.5, 111.2, 21.9. MS (ESI) *m/z* 261.1 [MH^+^].

### 7-Bromo-3-methoxypyrido[1,2-*a*]benzimidazole (9)

The same reaction described above to prepare **6** was used, and 223 mg of **9** was obtained in a yield of 8.05%. 1H NMR (400 MHz, CDCl_3_) δ 8.21 (d, *J* = 7.5 Hz, 1H), 7.95 (d, *J* = 1.7 Hz, 1H), 7.63 (d, *J* = 8.7 Hz, 1H), 7.38 (dd, *J *= 8.4, 1.7 Hz, 1H), 6.87 (d, *J* = 2.3 Hz, 1H), 6.59 (dd, *J* = 7.5, 2.6 Hz, 1H), 3.93 (s, 3H). ^13^C NMR (100 MHz, CDCl_3_) δ 161.5, 151.4, 146.4, 127.6, 125.4, 123.1, 121.6, 118.1, 110.7, 106.5, 93.9, 55.7. MS (ESI) *m/z* 277.1 [MH^+^].

### 7-Bromo-3-dimethylaminopyrido[1,2-*a*]benzimidazole (10)

2-Bromo-*N,N*-dimethylpyridin-4-amine (**5**) was synthesized according to the method reported previously^28^. Using **5**, the same reaction described above to prepare **6** was used, and 94.9 mg of **10** was obtained in a yield of 6.00%. ^1^H NMR (400 MHz, CDCl_3_) δ 8.16 (d, *J* = 7.5 Hz, 1H), 7.86 (s, 1H), 7.56 (d, *J* = 8.7 Hz, 1H), 7.30 (d, *J* = 8.7 Hz, 1H), 6.60 (d, *J* = 7.5 Hz, 1H), 6.55 (s, 1H), 3.14 (s, 6H). ^13^C NMR (100 MHz, CDCl_3_) δ 152.5, 150.8, 147.0, 127.7, 124.8, 121.5, 120.5, 117.8, 110.1, 102.3, 90.8, 40.0. MS (ESI) *m/z* 290.1 [MH^+^].

### 7-Tributylstannyl-3-phenylpyrido[1,2-*a*]benzimidazole (11)

A mixture of **6** (95.0 mg, 0.294 mmol), bis(tributyltin) (295 μL, 0.588 mmol), and (Ph_3_P)_4_Pd (146 mg, 0.126 mmol) in a mixed solvent (36.0 mL, dioxane/Et_3_N = 2/1) was stirred under reflux for 3 h. The solvent was removed, and the residue was purified by silica gel chromatography (ethyl acetate/hexane = 1/2) to give 26.7 mg of **11** (17.0%). ^1^H NMR (400 MHz, CDCl_3_) δ 8.48 (d, *J* = 7.3 Hz, 1H), 8.08 (s, 1H), 7.87–7.89 (m, 2H), 7.72 (d, *J* = 7.5 Hz, 2H), 7.44–7.53 (m, 4H), 7.12 (dd, *J* = 7.2, 1.7 Hz, 1H), 0.87–1.63 (m, 27H). ^13^C NMR (100 MHz, CDCl_3_) δ 148.5, 145.1, 142.0, 139.0, 138.4, 129.1, 128.8, 128.6, 128.3, 127.8, 126.9, 125.0, 114.4, 110.1, 110.0, 29.1, 27.4, 13.7, 9.8. MS (ESI) *m/z* 535.4 [MH^+^].

### 7-Tributylstannylpyrido[1,2-*a*]benzimidazole (12)

The same reaction described above to prepare **11** was used, and 510 mg of **12** was obtained in a yield of 32.8%. ^1^H NMR (400 MHz, CDCl_3_) δ 8.46 (d, *J* = 7.0 Hz, 1H), 8.08 (s, 1H), 7.88 (d, *J* = 8.1 Hz, 1H), 7.69 (d, *J *= 9.3 Hz, 1H), 7.45 (d, *J* = 8.1 Hz, 1H), 7.40–7.42 (m, 1H), 6.84 (dd, *J* = 7.0, 7.0 Hz, 1H), 0.87–1.64 (m, 27H). ^13^C NMR (100 MHz, CDCl_3_) δ 147.9, 144.4, 138.8, 129.1, 128.6, 128.2, 127.9, 125.0, 117.9, 110.0, 109.8, 29.0, 27.3, 13.6, 9.8. MS (ESI) *m/z* 459.3 [MH^+^].

### 7-Tributylstannyl-3-methylpyrido[1,2-*a*]benzimidazole (13)

The same reaction described above to prepare **11** was used, and 14.9 mg of **13** was obtained in a yield of 13.2%. ^1^H NMR (400 MHz, CDCl_3_) δ 8.31 (d, *J* = 7.0 Hz, 1H), 8.02 (s, 1H), 7.82 (d, *J* = 7.8 Hz, 1H), 7.38–7.42 (m, 2H), 6.66 (d, *J* = 7.0 Hz, 1H), 2.46 (s, 3H), 1.53–1.61 (m, 6H), 1.32–1.39 (m, 6H), 1.10–1.14 (m, 6H), 0.86–0.91 (m, 9H). ^13^C NMR (100 MHz, CDCl_3_) δ 148.6, 144.7, 140.5, 138.4, 128.7, 127.8, 127.6, 124.2, 116.0, 112.9, 109.6, 29.1, 27.4, 21.9, 13.7, 9.8. MS (ESI) *m/z* 473.3 [MH^+^].

### 7-Tributylstannyl-3-methoxypyrido[1,2-*a*]benzimidazole (14)

The same reaction described above to prepare **11** was used, and 47.9 mg of **14** was obtained in a yield of 23.9%. ^1^H NMR (400 MHz, CDCl_3_) δ 8.24 (d, *J* = 7.5 Hz, 1H), 7.95 (s, 1H), 7.75 (d, *J* = 7.5 Hz, 1H), 7.36 (d, *J* = 7.8 Hz, 1H), 6.86 (d, *J* = 2.3 Hz, 1H), 6.54 (dd, *J* = 7.2, 2.3 Hz, 1H), 3.93(s, 3H), 1.54–1.60 (m, 6H), 1.32–1.37 (m, 6H), 1.09–1.13 (m, 6H), 0.87–0.90 (m, 9H). ^13^C NMR (100 MHz, CDCl_3_) δ 161.0, 150.1, 145.1, 137.8, 128.7, 127.5, 126.9, 125.4, 109.2, 105.8, 93.9, 55.6, 29.1, 27.4, 13.7, 9.7. MS (ESI) *m/z* 489.3 [MH^+^].

### 7-Tributylstannyl-3-dimethylaminopyrido[1,2-*a*]benzimidazole (15)

The same reaction described above to prepare **11** was used, and 18.0 mg of **15** was obtained in a yield of 11.0%. ^1^H NMR (400 MHz, CD_3_OD) δ 8.41 (d, *J* = 7.5 Hz, 1H), 7.81 (d, *J* = 7.8 Hz, 1H), 7.66 (s, 1H), 7.24 (d, *J* = 7.5 Hz, 1H), 6.65 (dd, *J* = 7.8, 2.6 Hz, 1H), 6.32 (d, *J* = 2.3 Hz, 1H), 3.06 (s, 6H), 1.56–1.63 (m, 6H), 1.32–1.41 (m, 6H), 1.10–1.14 (m, 6H), 0.88–0.92 (m, 9H). ^13^C NMR (100 MHz, CDCl_3_) δ 151.2, 150.6, 137.3, 128.7, 126.3, 126.0, 124.8, 108.7, 101.9, 99.9, 91.2, 40.1, 29.1, 27.4, 13.7, 9.8. MS (ESI) *m/z* 502.4 [MH^+^].

### 7-Iodo-3-phenylpyrido[1,2-*a*]benzimidazole (16, BIP-Ph)

To a solution of **11** (24.7 mg, 0.0463 mmol) in chloroform (15.0 mL) was added a solution of iodine in chloroform (1.00 mL, 50 mg/mL) at room temperature. The mixture was stirred for 1.5 h, and saturated NaHSO_3_ aq. was added to quench the reaction. The mixture was extracted with chloroform (100 mL), and the organic phase was separated and dried over MgSO_4_. The solvent was removed and the residue was purified by silica gel chromatography (ethyl acetate/hexane = 1/2) to give **16** (10.3 mg, 60.2%). ^1^H NMR (400 MHz, DMSO-*d*_6_) δ 9.18 (d, *J* = 7.3 Hz, 1H), 8.19–8.22 (m, 2H), 7.93–7.99 (m, 3H), 7.66 (dd, J = 8.4, 1.4 Hz, 1H), 7.51–7.57 (m, 2H), 7.46–7.51 (m, 2H). ^13^C NMR (100 MHz, CDCl_3_) δ 149.1, 146.2, 143.2, 137.9, 129.7, 129.2, 129.1, 128.6, 127.9, 127.0, 125.0, 114.2, 111.9, 111.2, 90.0. HRMS (EI) *m/z* calcd. for C_17_H_11_IN_2_ (M^+^) 369.9967, found 369.9960.

### 7-Iodopyrido[1,2-*a*]benzimidazole (17, BIP-H)

The same reaction described above to prepare **16** was used, and 210 mg of **17** was obtained in a yield of 64.2%. ^1^H NMR (400 MHz, DMSO-*d*_6_) δ 9.10 (dd, *J* = 6.7, 0.9 Hz, 1H), 8.17–8.19 (m, 2H), 7.59–7.70 (m, 3H), 7.05 (dd, J = 6.7, 6.7 Hz, 1H). ^13^C NMR (100 MHz, CDCl_3_) δ 148.8, 146.1, 129.9, 129.5, 128.9, 128.1, 125.1, 118.1, 111.8, 110.8, 89.7. HRMS (EI) *m/z* calcd. for C_11_H_7_IN_2_ (M^+^) 293.9654, found 293.9660.

### 7-Iodo-3-methylpyrido[1,2-*a*]benzimidazole (18, BIP-Me)

The same reaction described above to prepare **16** was used, and 5.50 mg of **18** was obtained in a yield of 65.2%. ^1^H NMR (400 MHz, CDCl_3_) δ 8.29 (d, *J* = 7.0 Hz, 1H), 8.24 (d, *J* = 1.2 Hz, 1H), 7.60 (s, 2H), 7.43 (s, 1H), 6.72 (d, J = 7.2 Hz, 1H), 2.48 (s, 3H). ^13^C NMR (100 MHz, CDCl_3_) δ 149.2, 146.2, 141.5, 128.8, 128.4, 128.0, 124.1, 115.9, 113.5, 111.6, 89.3, 21.9. HRMS (EI) *m/z* calcd. for C_12_H_9_IN_2_ (M^+^) 307.9811, found 307.9808.

### 7-Iodo-3-methoxypyrido[1,2-*a*]benzimidazole (19, BIP-OMe)

The same reaction described above to prepare **16** was used, and 14.2 mg of **19** was obtained in a yield of 56.3%. ^1^H NMR (400 MHz, CDCl_3_) δ 8.20 (d, *J* = 7.5 Hz, 1H), 8.17 (d, *J* = 1.2 Hz, 1H), 7.51–7.57 (m, 2H), 6.86 (d, *J* = 2.3 Hz, 1H), 6.56 (dd, *J* = 7.5, 2.3 Hz, 1H), 3.93 (s, 3H). ^13^C NMR (100 MHz, CDCl_3_) δ 161.7, 151.0, 146.5, 128.8, 128.2, 127.8, 125.5, 111.2, 106.8, 94.0, 89.0, 55.8. HRMS (EI) *m/z* calcd. for C_12_H_9_IN_2_O (M^+^) 323.9760, found 323.9762.

### 7-Iodo-3-dimethylaminopyrido[1,2-*a*]benzimidazole (20, BIP-NMe_2_)

The same reaction described above to prepare **16** was used, and 5.50 mg of **20** was obtained in a yield of 16.7%. ^1^H NMR (400 MHz, CD_3_OD) δ 8.75 (d, *J* = 7.8 Hz, 1H), 7.94 (s, 1H), 7.85 (d, *J* = 8.7 Hz, 1H), 7.72 (d, *J* = 8.4 Hz, 1H), 7.08 (d, *J* = 7.8 Hz, 1H), 6.56 (s, 1H), 3.25 (s, 6H). ^13^C NMR (100 MHz, CDCl_3_) δ 152.1, 150.1, 147.5, 128.3, 127.1, 126.7, 124.8, 110.6, 102.4, 90.7, 88.6, 40.1. HRMS (EI) *m/z* calcd. for C_13_H_12_IN_3_ (M^+^) 337.0076, found 337.0074.

### Radiolabeling

The radioiodinated forms of ligands were prepared from the corresponding tributyltin precursors by iododestannylation. Briefly, to initiate the reaction, a tributyltin precursor (200 μg/200 μL EtOH) was added to a mixture of [^125^I]NaI (3.70–7.40 MBq, specific activity: 81.4 TBq/mmol) in 20 μL EtOH, 100 μL of 3% H_2_O_2_ aq., and 100 μL of 1 N HCl aq. in a sealed vial. The reaction to obtain radioiodinated ligands was allowed to proceed at room temperature for 40 min and terminated by the addition of saturated NaHSO_3_ aq. (200 μL). After neutralization with sodium hydrogen carbonate and extraction with ethyl acetate, the organic phase was dried by passing through a column filled with anhydrous Na_2_SO_4_. The solution was gas-dried with a stream of nitrogen gas. The crude radioiodinated ligands were purified by HPLC on a COSMOSIL 5C_18_-AR-II column with an isocratic solvent of acetonitrile/H_2_O (with or without TFA) at a flow rate of 1.0 mL/min.

### Measurement of log P values

The partition coefficients of each radioiodinated ligand were determined according to the conventional method^25^.

### *In vitro* autoradiography

The presence and location of tau and Aβ deposits in the AD brain sections were confirmed with immunohistochemical staining using an anti-phosphorylated tau antibody (AT8) and Aβ_1–42_ antibody (BC05), respectively (See the Supporting Information). [^125^I]IMPY was synthesized according to the method reported previously^32^. Six-micrometer-thick serial sections of paraffin-embedded blocks were used for staining. The sections were subjected to two 15-min incubations in xylene, two 1-min incubations in 100% EtOH, one 1-min incubation in 90% EtOH, and one 1-min incubation in 70% EtOH to completely deparaffinize them, followed by two 2.5-min washes in water. The sections were incubated with radioiodinated ligands (370 kBq/mL in 10% or 50% EtOH) for 2 h at room temperature. They were then dipped in 50% EtOH for 1 h and washed with H_2_O for 1 min. After drying, the ^125^I-labeled sections were exposed to a BAS imaging plate (Fuji Film) overnight. Autoradiographic images were obtained using a BAS5000 scanner system (Fuji Film). A two-tailed, unpaired t-test, with Welch’s correction as necessary, was performed to determine the significance of the difference in radioactivity accumulation in the gray matter between the frontal and temporal lobes. Statistical analyses were performed with GraphPad Prism 5 (GraphPad Software, Inc.).

### *In vitro* blocking study

We used the same postmortem brain tissues as employed for *in vitro* autoradiography. The sections were incubated with [^125^I]BIP-NMe_2_ (370 kBq/mL in 10% EtOH) for 2 h at room temperature in the presence or absence of nonradioactive BIP-NMe_2_ (1 or 10 μM). After incubation, we dipped the sections and exposed them to a BAS imaging plate with the same methods as *in vitro* autoradiographic studies. The autoradiographic images were obtained using a BAS5000 scanner system.

### Brain uptake and clearance in normal mice

A saline solution (100 μL) of each [^125^I]BIP derivative (19.6-29.4 kBq) containing ethanol (10.0 μL) and Tween 80 (0.100 μL) was injected intravenously directly into the tails of ddY mice (5 weeks old, male). The mice were sacrificed at various time-points postinjection. The brains were removed and weighed, and the radioactivity was measured with an automatic γ counter (Wallac WIZARD 1470, PerkinElmer).

### Analysis of radiometabolites in blood and brain

We performed the HPLC analyses of radiometabolites in the blood and brain according to a previously reported method^26^. [^125^I]BIP-NMe_2_ (1.11–1.48 MBq in 150 μL) was injected into the tail vein of ddY mice (n = 4 for each time point for the blood and brain). The mice were sacrificed at 2 and 10 min postinjection. Bood samples were obtained and centrifuged at 4,000 × *g* for 5 min at 4 °C. The plasma (200 μL) was separated and transferred to a tube containing acetonitrile (200 μL). The mixture was stirred in precipitate from the aqueous phase. The brain was removed from the mice at 2 and 10 min postinjection of [^125^I]BIP-NMe_2_, and homogenized in test tubes containing 500 μL of tris-buffered saline with a homogenizer (POLYTRON PT 10–35, KINEMATICA). Acetonitrile (500 μL) was added to a tube containing homogenized brain tissue and centrifuged at 4,000 × *g* for 5 min at 4 °C. The supernatants of the plasma and brain homogenates were filtrated using a 0.45-μm filter (Millipore). Then, the filtrate was analyzed using HPLC on a COSMOSIL 5C_18_-AR-II column with an isocratic solvent of acetonitrile/H_2_O/TFA (25/75/0.1) at a flow rate of 1.0 mL/min. The eluent was collected with a fraction collector (Frac-920, GE Healthcare) at 30-s intervals, and the radioactivity in each fraction (500 μL) was measured with an automatic γ counter (Wallac WIZARD 1470).

## Additional Information

**How to cite this article**: Ono, M. *et al*. Highly Selective Tau-SPECT Imaging Probes for Detection of Neurofibrillary Tangles in Alzheimer’s Disease. *Sci. Rep.* **6**, 34197; doi: 10.1038/srep34197 (2016).

## Supplementary Material

Supplementary Information

## Figures and Tables

**Table 1:** Ratio of radioactivity accumulation in the gray matter of the temporal lobe (a) against the gray matter of the frontal lobe (b).

Compound	a/b ratio
[^125^I]BIP-Ph	3.3
[^125^I]BIP-H	2.1
[^125^I]BIP-Me	23.9
[^125^I]BIP-OMe	12.2
[^125^I]BIP-NMe_2_	12.9
[^125^I]IMPY	1.8

**Table 2:** Brain uptake of ^125^I-labeled BIP derivatives after intravenous injection into normal mice^a^, molecular weight, and log P-value^b^ of ^125^I-labeled BIP derivatives.

Compound	% ID/g in brain			Ratio		Molecular weight	log P
	2 min	30 min	60 min	2 min/30 min	2 min/60 min		
[^125^I]BIP-Ph	3.51 ± 0.20	0.65 ± 0.06	0.23 ± 0.03	5.4	15.3	370	2.64 ± 0.03
[^125^I]BIP-H	4.74 ± 0.57	0.12 ± 0.01	0.06 ± 0.01	39.5	79.0	294	3.22 ± 0.04
[^125^I]BIP-Me	4.28 ± 0.50	0.14 ± 0.03	0.04 ± 0.00	30.6	107.0	308	3.37 ± 0.02
[^125^I]BIP-OMe	4.30 ± 0.41	0.17 ± 0.02	0.08 ± 0.03	25.3	53.8	324	2.71 ± 0.01
[^125^I]BIP-NMe_2_	3.98 ± 0.32	0.38 ± 0.03	0.16 ± 0.01	10.5	24.9	337	2.40 ± 0.05

^a^Each value represents the mean ± SD of five animals.

^b^Each value represents the mean ± SD of three experiments.
