# Supplementary material for: Highly Selective Tau-SPECT Imaging Probes for Detection of Neurofibrillary Tangles in Alzheimer’s Disease
Source: Sci Rep. 2016 Sep 30;6:34197. doi: 10.1038/srep34197 (PMC5043239; doi:10.1038/srep34197)
Supplement: Supplementary Information [file srep34197-s1.pdf]

Supporting Information for

Highly Selective Tau-SPECT Imaging Probes for Detection of Neurofibrillary Tangles in Alzheimer's Disease

Masahiro Ono*[,1], Hiroyuki Watanabe[1], Ayane Kitada[1], Kenji Matsumura[1], Masafumi Ihara[2], Hideo Saji[1]

[1]Department of Patho-Functional Bioanalysis, Graduate School of Pharmaceutical Sciences, Kyoto University

[2]Department of Stroke and Cerebrovascular Diseases, National Cerebral and Cardiovascular Center, 5-7-1 Fujishiro-dai, Suita, Osaka 565-8565, Japan.

*To whom correspondence should be addressed: Phone +81-75-753-4608, Fax: +81-75-753-4568, e-mail: ono@pharm.kyoto-u.ac.jp for M. Ono.

In vivo biodistribution in normal mice

The animal experiments were conducted in accordance with our institutional guidelines and approved by Kyoto University. A saline solution (100 μL) of each radioiodinated ligand (19.6-29.4 kBq) containing ethanol (10.0 μL) and Tween 80 (0.1 μL) was injected intravenously directly into the tails of ddY mice (5 weeks old, male). The mice were sacrificed at various time-points postinjection. The organs of interest were removed and weighed, and the radioactivity was measured with an automatic γ counter (Wallac WIZARD 1470, PerkinElmer).

Table S1. Biodistribution of radioactivity after intravenous injection of [^{125}I]BIP derivatives in normal mice[a]

Tissue	Time after injection (min)			
	2	10	30	60
		[^{125}I]BIP-Me		
Brain	4.28 (0.50)	0.94 (0.17)	0.14 (0.03)	0.04 (0.00)
Blood	3.63 (0.19)	2.37 (0.22)	1.53 (0.52)	0.77 (0.12)
Liver	19.7 (2.89)	13.7 (1.67)	7.32 (0.71)	4.69 (0.54)
Kidney	7.32 (0.61)	6.75 (0.83)	4.05 (1.19)	2.19 (1.23)
Intestine	3.38 (0.55)	14.2 (5.56)	19.5 (2.03)	28.7 (5.09)
Spleen	4.22 (0.43)	1.94 (0.29)	0.60 (0.13)	0.36 (0.10)
Pancreas	3.85 (0.39)	1.38 (0.29)	0.50 (0.08)	0.40 (0.24)
Heart	4.40 (0.59)	1.61 (0.19)	0.66 (0.10)	0.36 (0.07)

Lung	6.54 (0.76)	2.37 (0.31)	1.14 (0.11)	0.65 (0.11)
Thyroid[b]	0.06 (0.03)	0.03 (0.02)	0.02 (0.00)	0.01 (0.00)
Stomach[b]	2.68 (0.25)	6.52 (1.23)	7.05 (2.50)	7.43 (1.29)
		[^{125}I]BIP-OMe		
Brain	4.30 (0.41)	0.71 (0.14)	0.17 (0.02)	0.08 (0.03)
Blood	5.41 (0.40)	3.39 (0.12)	2.24 (0.27)	1.27 (0.15)
Liver	17.8 (3.98)	16.2 (1.80)	7.25 (1.28)	4.61 (0.35)
Kidney	8.66 (0.73)	7.38 (1.46)	5.74 (2.30)	2.20 (1.20)
Intestine	2.94 (0.15)	8.99 (1.43)	16.8 (4.30)	20.1 (4.03)
Spleen	3.60 (1.10)	3.16 (0.18)	1.07 (0.08)	0.63 (0.10)
Pancreas	4.38 (0.30)	1.59 (0.18)	0.86 (0.22)	0.53 (0.13)
Heart	5.17 (0.27)	1.94 (0.18)	0.99 (0.08)	0.52 (0.08)
Lung	6.09 (0.34)	3.33 (0.24)	1.76 (0.11)	1.21 (0.30)
Thyroid[b]	0.10 (0.05)	0.04 (0.02)	0.01 (0.00)	0.01 (0.00)
Stomach[b]	2.30 (0.33)	5.47 (0.88)	6.62 (1.28)	5.34 (1.23)
		[^{125}I]BIP-NMe_2		
Brain	3.98 (0.32)	1.66 (0.16)	0.38 (0.03)	0.16 (0.01)
Blood	2.91 (0.75)	1.48 (0.18)	1.18 (0.26)	0.93 (0.23)
Liver	9.78 (2.50)	18.1 (0.53)	12.7 (1.35)	5.82 (0.91)
Kidney	14.3 (1.91)	17.0 (1.70)	7.45 (1.11)	5.45 (1.90)
Intestine	3.07 (0.52)	7.45 (0.50)	14.5 (3.43)	23.5 (3.65)
Spleen	2.97 (0.92)	5.15 (0.84)	2.97 (0.44)	1.97 (0.25)
Pancreas	5.66 (1.03)	4.77 (0.27)	1.51 (0.19)	0.80 (0.15)
Heart	6.38 (1.09)	2.53 (0.24)	1.00 (0.15)	0.63 (0.05)

Lung	8.80 (1.70)	3.59 (0.43)	1.77 (0.31)	0.99 (0.07)
Thyroid[b]	0.05 (0.03)	0.03 (0.01)	0.02 (0.01)	0.01 (0.00)
Stomach[b]	1.71 (0.43)	3.30 (0.49)	6.35 (1.13)	7.59 (1.39)

[a] Each value represents the mean (SD) of 5 animals.

[b] Expressed as % injected dose per organ.

Immunohistochemical staining using brain sections from a patient with Alzheimer's disease

We conducted immunohistopathological staining according to a method reported previously. [1] Postmortem brain tissues from an autopsy-confirmed case of AD (a 76-year-old male) were obtained from the Graduate School of Medicine, Kyoto University. Six-micrometer-thick serial sections of paraffin-embedded blocks were used for staining. The sections were subjected to two 15-min incubations in xylene, two 1-min incubations in 100% EtOH, one 1-min incubation in 90% EtOH, one 1-min incubation in 80% EtOH, and one 1-min incubation in 70% EtOH to completely deparaffinize them, followed by two 2.5-min washes in water. They were then autoclaved for 15 min in 0.01 M citric acid buffer (pH 6.8) to activate the antigen. After two 5-min incubations in PBS-Tween 20, the sections were incubated at room temperature with an anti-phosphorylated tau (AT8, Thermo Scientific) or $A\beta_{1-42}$ (BC05, Wako) primary antibody for 1 h. After three 5-min incubations in PBS-Tween 20, they were incubated with biotinylated goat anti-mouse IgG (Histofine Simple Stain Mouse MAX-PO (MULTI), Nichirei Biosciences inc.) at room temperature for 30 min. After three 3-min incubations in PBS-Tween 20 and one 5-min incubation in TBS, the sections were incubated with DAB as a chromogen for 1 min. After being washed with water, the sections were observed under a microscope (BZ-9000, KEYENCE).

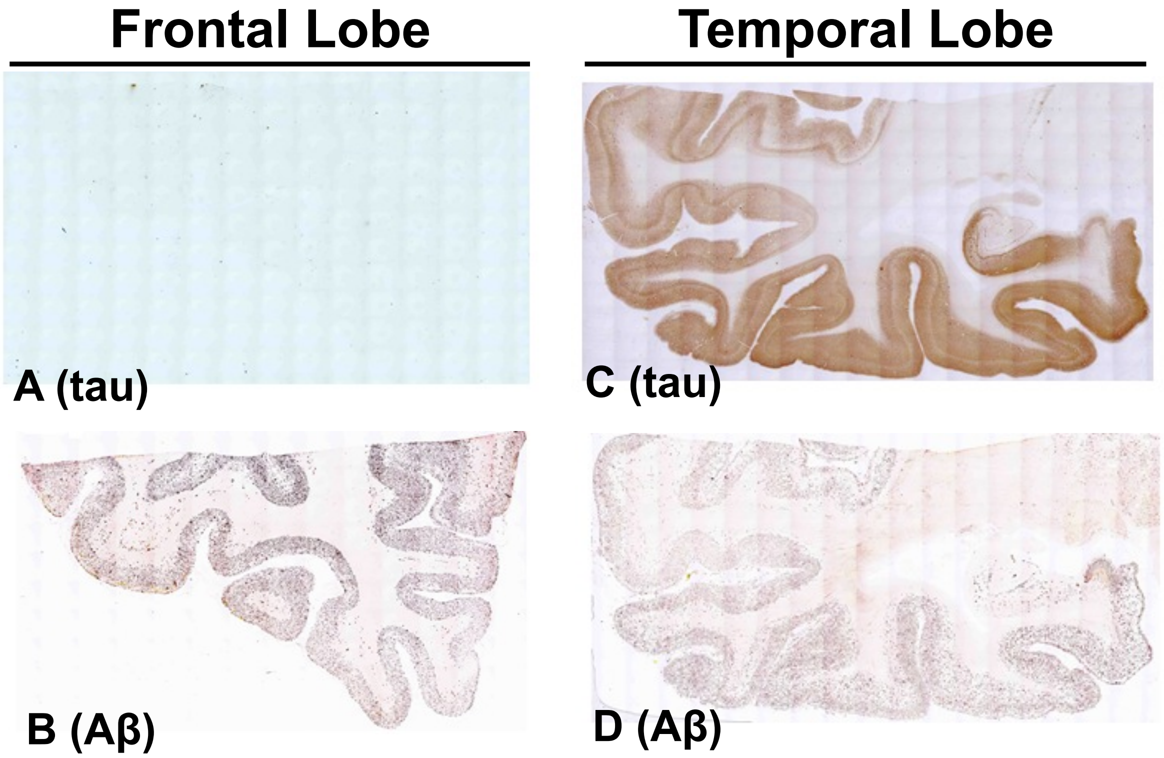

Figure S1. Immunohistochemical staining with antibodies against phosphorylated tau (AT8: A and C) and $A\beta_{1\text{–}42}$ (BC05: B and D) in sections of the frontal lobe (A and B) and temporal lobe (C and D) from an AD patient (76-year-old male). Adapted from reference 1.

Enlarged *in vitro* autoradiograms with [^{125}I]IMPY and [^{125}I]BIP-NMe$_2$

We carried out in vitro autoradiography of AD brain sections with [^{125}I]IMPY and [^{125}I]BIP-NMe$_2$ using a similar method described in the text.

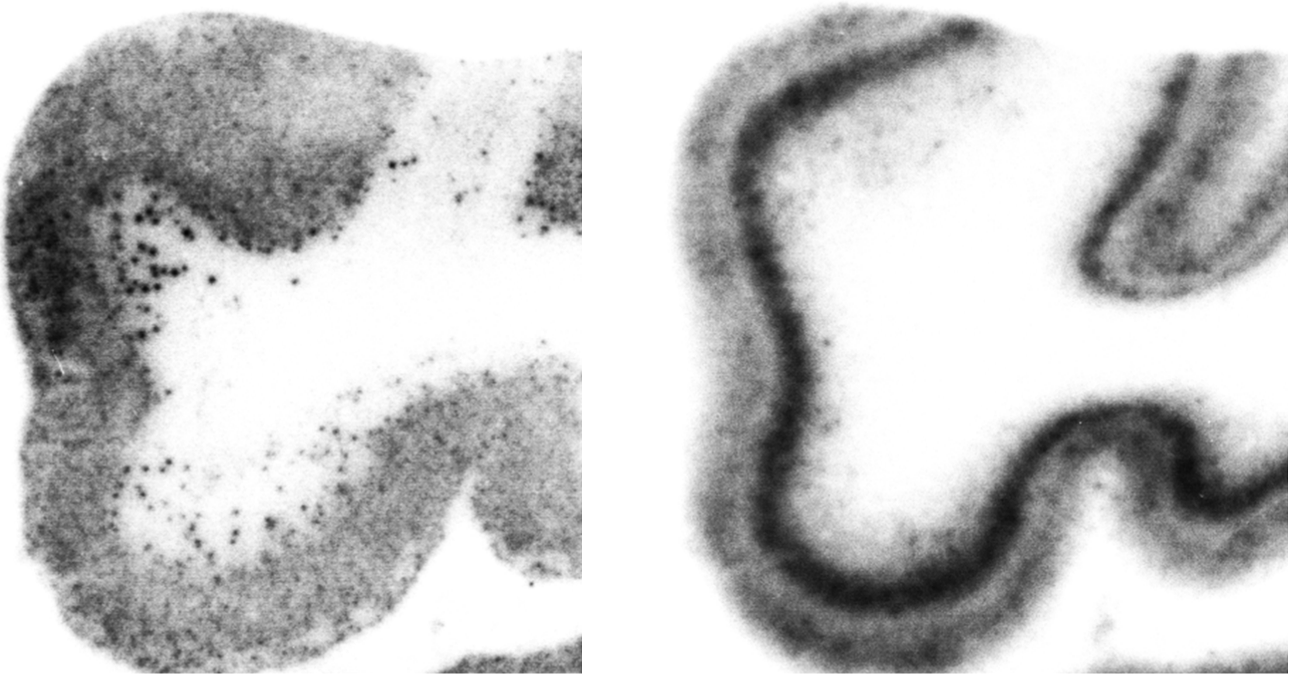

[^{125}I]IMPY [^{125}I]BIP-NMe$_2$

Figure S2. Representative *in vitro* autoradiograms with [^{125}I]IMPY and [^{125}I]BIP-NMe$_2$ on AD brain sections from temporal lobes.

***In vitro* stability evaluation in mouse plasma.**

We evaluated *in vitro* stability in mouse plasma according to a method reported previously[1]. Five-week-old mice (male, ddy, 25–28 g) were anesthetized with isoflurane. Blood samples were collected from the hearts and centrifuged (10 min, 4,000 X *g*). The supernatant was collected and 20.0 μL of [^{125}I]BIP-NMe$_2$ was added to 200 μL of mouse serum. The sample was incubated at 37 °C for 60 min. After the incubation, 400 μL of acetonitrile was added to the sample, and it was

centrifuged (10 min, 4,000 X *g*). The supernatant was collected and filtered with a Cosmonice Filter (S) (0.45 μm, 4 mm), and the filtrate was analyzed by HPLC.

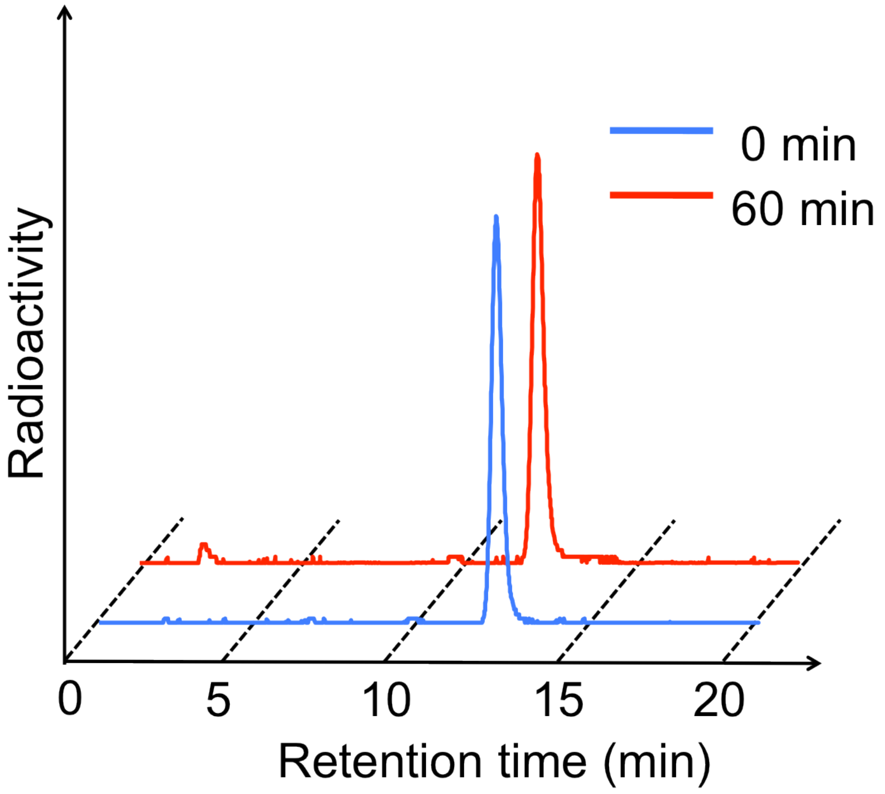

Figure S3. HPLC analysis of [^{125}I]BIP-NMe_2 in mouse plasma before (blue line) and after the incubation for 60 min (red line) at 37 °C.

Reference

1. Matsumura K, *et al*. Structure-Activity Relationship Study of Heterocyclic Phenylethenyl and Pyridinylethenyl Derivatives as Tau-Imaging Agents That Selectively Detect Neurofibrillary Tangles in Alzheimer's Disease Brains. *J Med Chem* **58**, 7241-7257 (2015).
